# Meetings of Societies

**Published:** 1939

**Authors:** 


### Meetings of Societies

### Bristol Medico-Chirurgical Society.

SIXTY-SIXTH SESSION, 1938–1939.

THE fourth meeting of the Session was held in the Physics Lecture Theatre, Royal Fort, on Wednesday, 11th January, 1939, at 8.30 p.m.

Dr. Leonard Moore was in the Chair, and sixty-nine members were present.

Mr. F. Willway showed skiagrams illustrating intracranial lesions and method for investigation and localization, and Dr. C. H. G. Price showed pathological specimens.

Professor Gilbert Strachan gave a very practical paper dealing with "**Obstetrical Emergencies.**" A lively discussion followed in which Dr. Leonard Moore, Professor Smythe, Dr. Phillips and Mr. Nicholson Lailey took part, and Professor Strachan replied to the questions.

---

The fifth meeting of the session was held in the Physics Lecture Theatre, Royal Fort, on Wednesday, 8th February, 1939, at 8.30 p.m.

Dr. Leonard Moore was in the Chair, and 106 members were present.

Dr. Bergin showed skiagrams, and Professor Hewer showed pathological specimens.

Mr. W. Heneage Ogilvie gave a lecture entitled "**Recent Advances in Surgery,**" distinguishing clearly between what is merely " recent " and what is truly an " advance." Professor Rendle Short proposed, and Mr. Adams seconded a vote of thanks to Mr. Ogilvie for his very helpful survey of many branches of surgery.

The sixth meeting of the session was held in the Physics Lecture Theatre, Royal Fort, on Wednesday, 8th March, 1939, at 8.30 p.m.

Dr. Leonard Moore was in the Chair, and forty-four members were present.

Dr. Bush showed skiagrams, and Dr. Fraser showed pathological specimens.

Mr. Anthony Palin then gave an address on "**Some Recent Advances in Ophthalmology,**" with special reference to the use of contact glasses, and to orthoptics in the treatment of squint. He showed a number of very interesting exhibits illustrating different types of work which ophthalmic surgery now embraces.

Mr. Ramsay Garden, and Mr. Coote took part in a discussion, and Mr. Palin replied to the questions.

---

The seventh meeting of the session was held in the Physics Lecture Theatre, Royal Fort, on Wednesday, 12th April, 1939, at 8.30 p.m.

Dr. Leonard Moore was in the Chair and sixty-two members were present.

Dr. A. L. Taylor showed pathological specimens, and Dr. Bush showed a short cine film illustrating a new radiographic method whereby different depths in the body were brought successfully into focus, in a series of X-ray photographs.

Dr. Bush explained the process and showed many very interesting examples of the value of this new method.

Dr. Nixon then read a paper on "**Diet in Pregnancy and Lactation.**" Mr. Shepherd, Professor Smythe, Mr. Palin, Dr. Phillips, Dr. Kemm and Dr. Richard Clarke took part in the discussion which followed, and Professor Nixon replied to questions raised.

Dr. Beryl Corner then read a paper on "**Some Aspects of Bronchography as an aid to the Diagnosis of Intrathoracic Disease,**" and showed a film which she had produced. The film showed the technique and the uses of this method of investigation. Dr. Beryl Corner also showed an excellent series of bronchographs which she had made of cases coming under her care.

Dr. Bush asked questions and Dr. Beryl Corner replied.

### British Medical Association.

### Bath, Bristol and Somerset Branch.

EIGHT meetings were held under the able presidency of Mr. Hubert Chitty. In his inaugural address the President gave his audience a most interesting account of his experiences in a number of tuberculous conditions that he had encountered during his many years of active surgical work, and this was illustrated with many excellent lantern slides. Papers were also given in Bristol by Mr. A. L. Eyre-Brook, who dealt with his recent visit to orthopædic hospitals in the U.S.A., and by Dr. A. W. Spence, who gave an account of recent advances in hormone therapy. The annual clinical meeting was held at the General Hospital. The attendance of both patients and practitioners was greatly impeded by the inclemency of the weather, as it snowed hard during the afternoon and evening of the day selected. At Bath, Dr. Barnes Burt gave a paper upon "**The Causation and Treatment of Brachial Neuritis.**" Dr. Wyllie also visited the Branch at Bath and read a paper upon "**The Diagnosis in Pyrexia of Obscure Origin in Children.**" Dr. Reginald Miller lectured at Taunton on the subject of the "**Medical Appendix,**" which produced a most interesting discussion : and at Weston-super-Mare, Dr. Norman Burgess gave an address upon "**The Seborrhoeic Dermatoses,**" published in this issue.

*Officers for* 1939–40.—President, Dr. G. McLeod ; Vice-Presidents, Mr. Hubert Chitty, F.R.C.S., Dr. A. J. H. Iles ; Treasurer, Dr. F. J. Gomez ; Secretary, Dr. C. H. G. Price.

